# Regulation of Basal Lateral Membrane Mobility and Permeability to Divalent Cations by Membrane Associated-Protein Kinase C

**DOI:** 10.1371/journal.pone.0080291

**Published:** 2013-11-08

**Authors:** Chao Zhang, Yuanyuan Zheng, Lihong Chen, Min Chen, Shenxuan Liang, Mosi Lin, Dali Luo

**Affiliations:** Department of Pharmacology, School of Chemical Biology & Pharmaceutical Sciences, Capital Medical University, Beijing, P.R. China; Cornell University, United States of America

## Abstract

Biological membrane stabilization is essential for maintenance of cellular homeostasis, functionality and appropriate response to various stimuli. Previous studies have showed that accumulation of PKCs in the cell membrane significantly downregulates the membrane fluidity and Ca^2+^ influxes through the membranes in activated cells. In addition, membrane-inserted form of PKCs has been found in a variety of resting mammalian cells and tissues. This study is aimed to investigate possible role of the endogenous membrane-associated PKCs in the modulation of basal membrane fluidity. Here, we showed that interfering PKC expression by chronic activation of PKC with phorbol myristate acetate (PMA) or shRNA targeting at PKCα lowered the levels of PKCα in cytosol, peripheral membrane and integral membrane pools, while short-term activation of PKC with PMA induced accumulation of PKCα in the membrane pool accompanied by a dramatic decrease in the cytosol fraction. The lateral membrane mobility increased or decreased in accordance with the abundance alterations in the membrane-associated PKCα by these treatments. In addition, membrane permeability to divalent cations including Ca^2+^, Mn^2+^ and Ba^2+^ were also potentiated or abrogated along with the changes in PKC expression on the plasma membrane. Membrane stabilizer ursodeoxycholate abolished both of the enhanced lateral membrane mobility and permeability to divalent cations due to PKCα deficiency, whereas Gö6983, a PKC antagonist, or Gd^3+^ and 2-aminoethyoxydipheyl borne, two Ca^2+^ channels blockers, showed no effect, suggesting that this PKC-related regulation is independent of PKC activation or a modulation of specific divalent cation channel. Thus, these data demonstrate that the native membrane-associated PKCα is involved in the maintenance of basal membrane stabilization in resting cells.

## Introduction

Protein kinase C (PKC) represents a family of serine-threonine protein kinases that relay multiple extracellular stimuli into intracellular effectors, thereby initiate or maintain various cellular functions and cell proliferation and survival. More than ten members of the PKC family have been identified by molecular cloning and are grouped into three major classes based on structural and ligand-binding differences in the regulatory domain: conventional, Ca^2+^-dependent PKCs (α, βI, βII, and γ), novel, Ca^2+^-independent PKCs (δ, ε, θ, and μ) and atypical, Ca^2+^- and lipid-independent PKCs (λ/ι and ζ) [1,2]. All of them exhibit differential tissue distributions, and different subcellular localizations and substrate specificities [3–6]. It is well accepted that PKCs are soluble and peripheral membrane proteins and translocate into membranes, including plasma membrane and endoplasmic reticulum upon activation [3–6]. Interestingly, several studies *in vitro* and *in vivo* have demonstrated a membrane-inserted form of all ten PKC isoforms in a variety of mammalian cells and tissues, which are termed integral PKCs [7–10]. Integral membrane proteins are different from soluble and peripheral membrane proteins in that a disruption of lipid bilayer is required for release of an integral membrane protein, whereas other fractions of proteins can be released with the lipid bilayer intact [7,11,12]. Zhu Y and Duan W have identified that the integral membrane PKC and peripheral membrane PKCs are 0.4–3% and 10–20% proportion of total cellular PKCs in resting state, and increased to 80 and 10 folds, respectively, in response to activation [7]. This dramatic translocation of PKCs from cytosol to membrane reflects a necessity of membrane-linked PKCs in their functional performance upon activation [1,2]. Likewise, the small amount of membrane PKCs existed in resting cells might also play a role in the maintenance of basal PKC activity, although their functional role in this loci is not understood yet [7]. 

Our previous data have showed that activation of PKC induces robust redistribution of isoform PKCα in the plasma membrane and endoplasmic reticulum from the cytosol, resulting in reductions of membrane fluidities and permeability to Ca^2+^ fluxes [13,14]. As biological membranes do not only confine compartments, but also control all communications between the interior and exterior of cells, including the transport of ions or molecules across membranes by means of specific transport proteins in or on membranes. Additionally, some components of the membrane, such as lipid and protein have been implicated to regulate ions or molecules transport between the intracellular and extracellular spaces [15–17]. Thus, we hypothesized that the native integral/peripheral membrane PKCs may participate in the regulation of basal cell membrane stabilization in resting status. 

We solve this issue by interfering PKCα and PKCβ expression in HEK293 cells, the most common isotypes in various types of cells, with shRNA-mediated silencing gene approach and persistent PKC activation that consequently causes robust downregulation of PKC expression [7,18,19], and evaluating the basal cell lateral membrane mobility, membrane permeability to divalent cations and the corresponding levels of PKC in different fractioned lysates. We find that the membrane-associated PKCα, especially the integral PKCα, is involved in the maintenance of cellular lateral membrane mobility and permeability to ions in resting cells.

## Materials and Methods

### 1 Materials

Fura-2/AM and DiIC_16_ (3) were purchased from Molecular Probes (Life Technologies Corporation, Shanghai, China). Anti-PKC isoform-specific antibodies and the shRNA plasmid transfection reagent (sc-108061) were purchased from Santa Cruz Biotechnology, Inc (Santa Cruz, Beijing, China). ShRNA plasmids targeting PKCα and PKCβ were purchased from Invitrogen (Life Technologies Corporation, Shanghai, China).Unless otherwise indicated, all other reagents and antibodies were obtained from Sigma-Aldrich (Shanghai, China). 

### 2 Cell culture

HEK293 cells obtained from ATCC were cultured at 37°C in Dulbecco’s modified Eagles medium (DMEM) containing 10% fetal bovine serum and 2 mM glutamine in humidified 5% CO_2_ and 95% air incubators. For Ca^2+^ measurements, HEK293 cells grown to about 80% confluence were detached with 0.125% trypsin and centrifuged for 5 min at 700 × g. HEK293 cells were washed with HEPES buffered physiological saline solution (HBSS in mM: NaCl 120, KCl 5.4, Mg_2_SO_4_ 0.8, HEPES 10, CaCl_2_ 1.8, glucose 10; pH 7.4, adjusted with NaOH). 

### 3 Ca^2+^ fluorescence measurements

Fluorescence measurements of [Ca^2+^]_i_ in HEK293 were performed as previously described [13,20]. In brief, the cells were loaded with 1 μM Fura-2/AM in the dark for 25 min at 37°C and then washed with Ca^2+^-HBSS. Then, the density of the cells was adjusted to 1×10^6^/ml with HBSS and the Fura-2 fluorescence changes in the cell suspension were measured under constant stirring at 37°C at excitation wavelengths of 340 nm and 380 nm and an emission wavelength of 510 nm using a fluorescence spectrophotometer (Hitachi, F7000). The changes of the Fura-2 fluorescence expresses as the ration of 340/380nm and sometime the concentration of Ca^2+^.

### 4 Mn^2+^ quench and Ba^2+^ entry measurements

As previously described [21], Mn^2+^ quench experiments were carried in nominally Ca^2+^-free HBSS with 0.1 mM MnCl_2_. Ftot represented the percentage of Fura-2 fluorescence quenched by Mn^2+^. 

Ba^2+^ influx experiments were performed with 1 mM BaCl_2_ in nominally Ca^2+^-free medium. Ba^2+^, the same as Ca^2+^, makes F340 increase and F380 decrease. Thus, the ratio of Fura-2 fluorescence demonstrates Ba^2+^ uptake. 

### 5 FRAP method

The measurement of lateral membrane mobility was performed using fluorescence recovery after photobleaching (FRAP) method as previously described [13], and two values, the mobile fraction (M_f_) and the diffusion constant (D) were adopted to indicate the variations of the lateral membrane mobility [22]. 

### 6 Preparation and transfection of shRNA plasmids targeting isoforms of PKC

The HEK293 cells were transfected with shRNA plasmids according to the transfection reagent instruction. Firstly, the optimal shRNA plasmid transfection reagent ratio was determined to be 1:3 (μg: μl) and the optimal shRNA plasmid DNA was 3 μg per 60 mm dish. Before transfection, the HEK293 cells were grown to 50~60% confluence in DMEM without Penicillin-Streptomycin for 24 h. The transfection reagent and shRNA plasmids were diluted and incubated in DMEM for 30min, and adjusted to the optimal concentration prior to transfection. Then cells were transfected with equal amounts of the luciferase (shCon), PKCα, and PKCβ shRNA plasmids at 37°C in a CO_2_ incubator for 6 h. After removing the transfection solution, the cells were cultured under normal growth conditions for 48 h before all experimental measurements were performed. 

### 7 Subcellular fractionation isolation

Total plasma membranes were prepared according to the procedure described by Zhu Y [7] with some modifications. Briefly, cells were lysed in isotonic buffer without detergent (20 mM Tris-HCl (pH 7.5), 150 mM NaCl, 10 mM EDTA, 5 mM EGTA, 20 mM NaF, 5 mM sodium pyrophosphate, 1 mM sodium vanadate and a cocktail of protease inhibitors) by a Dounce homogenizer, and 100 strokes were made until more than 90% cells were disrupted observed under microscope. After the lysate was centrifuged at 1000 × g for 10 min at 4°C to ensure plasma membrane purity, the supernatant was further centrifuged at 300,000 × g for 1 h at 4°C, and the pellet was subjected to further extraction for peripheral and integral membrane proteins, while the supernatant after the second centrifugation was regarded as the cytosolic fraction. 

Integral membrane proteins were separated from peripheral proteins by the nonionic detergent Triton X-114 phase separation [23]. The pellet (total cell membrane) was redissolved in 2% Triton X-114 buffer (mM) (pH 7.4, NaCl 150, Tris-HCl 10, EGTA 1, 2% (V/V) Triton X-114 and a cocktail of protease inhibitors), and incubated for 15 min at 4°C. The homogeneous solution appeared cloudy after incubating at 37°C for 3 min, which was then centrifuged for 1 min at 10,000 × g at room temperature. The peripheral proteins were in the upper (aqueous) phase, while the integral membrane proteins were recovered in the detergent phase. Both of the phases were re-extract to ensure purity, and the detergent phase was diluted with 100 μl TENT-OG buffer (mM: NaCl 150, Tris-HCl 25, EGTA 5, Octyl-β-D-glucopyranoside 60, 1% (V/V) Triton X-100 and a cocktail of protease inhibitors, pH 8.0). To concentrate proteins in cytosolic fraction and detergent/aqueous phases, solutions were lyophilized for 48 h at -80°C. Powders after lyophilization were diluted with water containing protease inhibitors for cytosolic fraction 500 μl, detergent phase 50 μl and aqueous phase 50 μl, which used for Western blotting analysis. 

### 8 Western blotting

The HEK293 cells lysates and the 3 different fractions of cells were blotted for detection of isotypes of PKC as previously described [13]. PKCα or PKCβ lysates (15 or 30 μg) from cells exposed to each type of shRNA-mediated interference, cytosol fraction (2% or 15 μg), peripheral membrane fraction (5% or 15 μg) and integral membrane fraction (5% or 15 μg) were heated for 5 min, resolved on a 10% SDS-PAGE gel, and transferred to PVDF membranes. Two rabbit anti-PKC antibodies were used to immunoblot proteins, one that binds strongly to PKCα (1:1500) and the other that detects PKCβ (1:1000). The primary antibody used for β-actin and GAPDH were mouse polyclonal anti-β-actin antibody (1:2000) and mouse polyclonal anti-GAPDH antibody (1:2000). Immunoreactive bands were detected using enhanced chemiluminescence and the intensity of each band was normalized with β-actin. 

### 9 Fluorescence staining

After PKCα shRNA transfected for 48 h or PMA short/long activation, HEK293 cells were washed three times with HBSS, fixed with phosphate buffer solution (PBS, pH=7.4) containing 4% formaldehyde for 10 min and permeabilized with 0.1% Triton X-100 in PBS. The anti-PKCα antibodies were used at a dilution of 1:100 and the secondary antibody Alexa Fluor 488-labeled goat anti-rabbit (Invitrogen) used at a dilution of 1:500. Hoechst 33258 (1 μg/ml, 5 min) was used to label the nucleus. The laser-scanning confocal microscope was used to detect the chemifluorescent as described [24].

### 10 Statistics

SPSS Statistics 13.0 software was used to test statistical significance. The 2-way paired or unpaired Student’s *t* tests were used for paired analysis between two groups. Data are presented as means ± S.D. and significance was set at p<0.05. QuantityOne software (Bio-Rad) was applied to analyze the band intensities in Western blot at the same time point.

## Results

### 1 Effect of PKC interference on plasma lateral membrane mobility in resting cells

**Figure 1:**
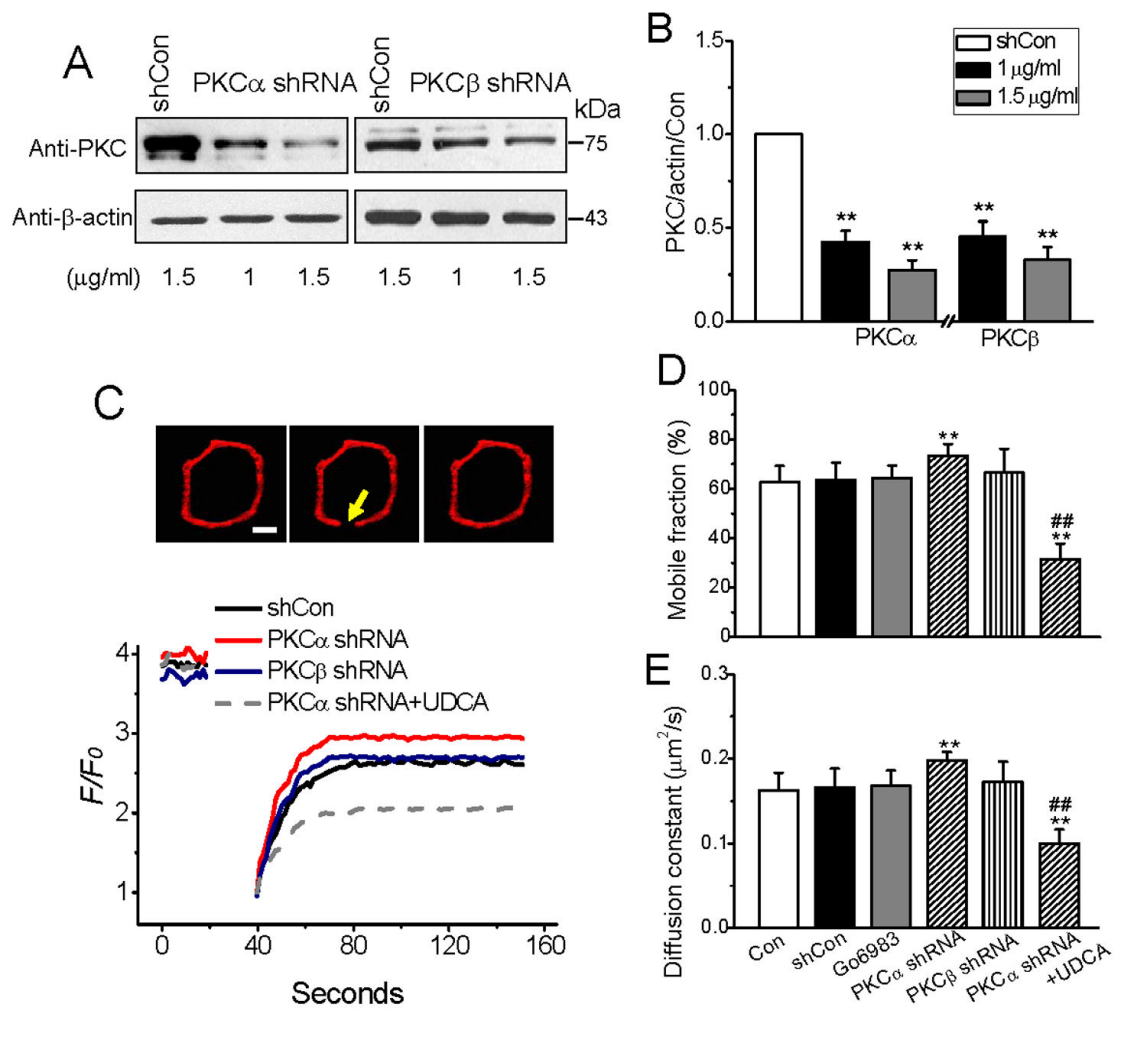
Effect of PKC interference on plasma membrane (PM) fluidity in resting cells. (A and B), Efficiency of RNA interference of PKC isoforms in HEK293 cells. Cells were transfected with PKCα and PKCβ shRNA, respectively, to knockdown PKCα and PKCβ, and expression levels of PKCα and PKCβ were detected by Western blotting in 3 separate experiments. (C−E), FRAP was used to detect PM fluidity change due to PKC knockdown. Typical fluorescence recovery images and realtime fluorescence recovery curves represent in 8–10 separate experiments, scale bar: 5 μm. Statistical analyses of the two measures: mobile fraction and diffusion constant (D and E), indicating the degree and the speed of fluorescence recovery, respectively, for comparing the effect of PKC knockdown on PM fluidity with control. **represent p<0.01 vs. the levels of PKC expression or the lateral membrane mobility in shCon-treated cells, respectively, and ^##^stands for p<0.01 vs. the levels without UDCA in PKCα-knockdown cells.

To address the issue of membrane-associated PKC likely regulating the membrane fluidity, we first determined the abundance of PKC expression in HEK293 cells interfered with two shRNA plasmids aimed at PKCα and PKCβ, respectively. Using Western blot approach with specific antibody for PKCα or PKCβ, we found a plasmid dose-dependent knockdown of PKCα and PKCβ expression in the cells transfected for 48 h, and approximate 70% reductions in both PKCα and PKCβ compared with that in shRNA vector transfected (shCon) cells, were obtained by 1.5 μg/ml plasmids (Figure 1A and B). Thus, the concentration of 1.5 μg/ml plasmids was adopted in the following tests.

The lateral cell membrane mobility was analyzed by FRAP method using DiIC_16_ (3) [22,25], and a membrane stabilizer ursodeoxycholate sodium salt (UDCA) [15,16] was used as control agent. As showed in Figure 1C–E, HEK293 cells deficient in PKCα demonstrated a significant increase in fluorescence recovery after photobleaching the cell membranes, a suggestive of mobility increase, whereas the PKCβ-knockdown cells and cells pretreated with Gö6983, a PKC inhibitor that inhibits both PKCα and PKCβ isoforms [26], showed no significant effect. UDCA (100 μM) alone reduced the plasma membrane mobility strikingly in native cells as previous reports [15,16] and also in the cells interfered with PKCα shRNA. 

### 2 Effect of PKC interference on basal Ca^2+^ influx in resting cells

**Figure 2:**
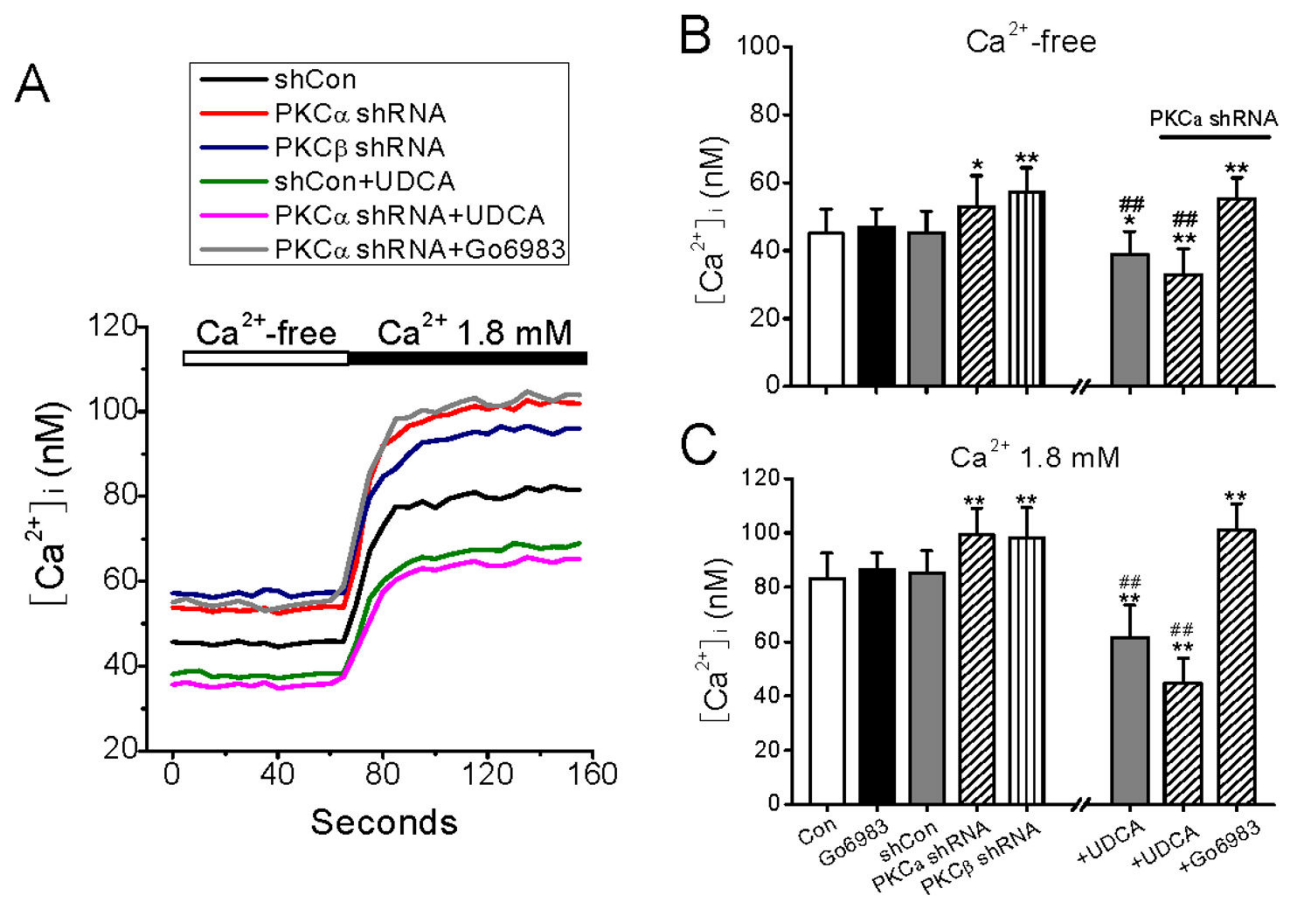
Effect of PKC interference on basal calcium in resting cells. (A), Typical traces illustrate basal [Ca^2+^]_i_ in resting HEK293 cells incubated in Ca^2+^-free medium, and following addition of 1.8 mM Ca^2+^ to the medium. The cells were transfected with PKCα and PKCβ shRNA, and pretreated with a membrane stabilizers UDCA (100 μM, 10 min). (B and C), The data in B and C represent the statistic results from separate groups as indicated in Ca^2+^-free and 1.8 mM Ca^2+^ medium, respectively. The Gö6983 (1 μM, 10min) was used to inhibit PKC. ^*^and ^**^represents p<0.05 and p<0.01 vs. the level of basal [Ca^2+^]_i_ in shCon-treated cells, and ^##^represent p<0.01 vs. level of shCon- or PKC shRNA-treated cells N=7−10 independent determinations for each bar.

Ca^2+^ is an important divalent cation in maintenance of basal cellular homeostasis and functions, and also in communicating signal transduction between the interior and exterior of the cells when they are stimulated. To examine whether the change in basal lateral membrane mobility due to PKC deficiency impacts the basal Ca^2+^ flux, we measured the intracellular Ca^2+^ concentration [Ca^2+^]_i_ in resting HEK293 cells incubated in Ca^2+^-free and then in 1.8 mM Ca^2+^-containing medium by Fura-2 indicator (Figure 2A). Indeed, HEK293 cells with PKCα or PKCβ knockdown demonstrated higher basal [Ca^2+^]_i_ in both Ca^2+^-free and 1.8 mM Ca^2+^-containing conditions than cells treated with shCon plasmid (Figure 2B and C). UDCA (100 μM) depressed the elevated [Ca^2+^]_i_ as well as the basal [Ca^2+^]_i_ levels in all groups of cells as it did in the lateral membrane mobility detection (Figure 1D and E). Although the amount of net elevated [Ca^2+^]_i_ was almost the same (~15% of that in shRNA cells) in both PKC isoform knockdown cells, most of the Ca^2+^ source was internal, i.e. leakage from Ca^2+^ stores, in PKCβ-knockdown cells, while the Ca^2+^ was from both internal and extracellular spaces in PKCα-knockdown cells. This difference in Ca^2+^ mobilization pathway was also corresponded with the observations that a detectable increase in the lateral membrane mobility was found in PKCα- but not in PKCβ-knockdown cells (Figure 1E), and consistent with previous report that PKCβ is much more important in regulating Ca^2+^ release than Ca^2+^ entry process [13]. Therefore, in the following experiments we mainly focused on the effect of PKCα on the plasma membrane permeability because it is feasible to be detected in intact cells at rest. When PKCs were blocked with Gö6983, no significant change was found in basal [Ca^2+^]_i_ under either Ca^2+^-free or 1.8 mM Ca^2+^ containing condition, indicating an independent of endogenous PKC activation in the regulation of basal Ca^2+^ fluxes. 

### 3 Effect of PKC interference on Mn^2+^ and Ba^2+^ influxes in resting cells

To clarify that the increased basal Ca^2+^ influx due to deficient PKCα is nonspecifically attributed to the changed lateral membrane mobility rather than a modulation of specific Ca^2+^ channel, Mn^2+^ and Ba^2+^ influxes in Ca^2+^-free medium were further monitored in response to PKCα interference. Mn^2+^ enters cells through divalent cation channels in plasma membrane, but quenches Fura-2 fluorescence at all wavelengths, while Ba^2+^ like Ca^2+^ gets into cells and produces an increase in the ratio (*F340/F380*) of Fura-2 fluorescence. It has been found that the entries of Ba^2+^ and Mn^2+^ into cells are sensitive to different agonists’ potentiations and different antagonists’ inhibitions, suggesting different divalent cation passages conducting their influxes [21,27,28]. However, the identity for each passage is unknown yet.

**Figure 3:**
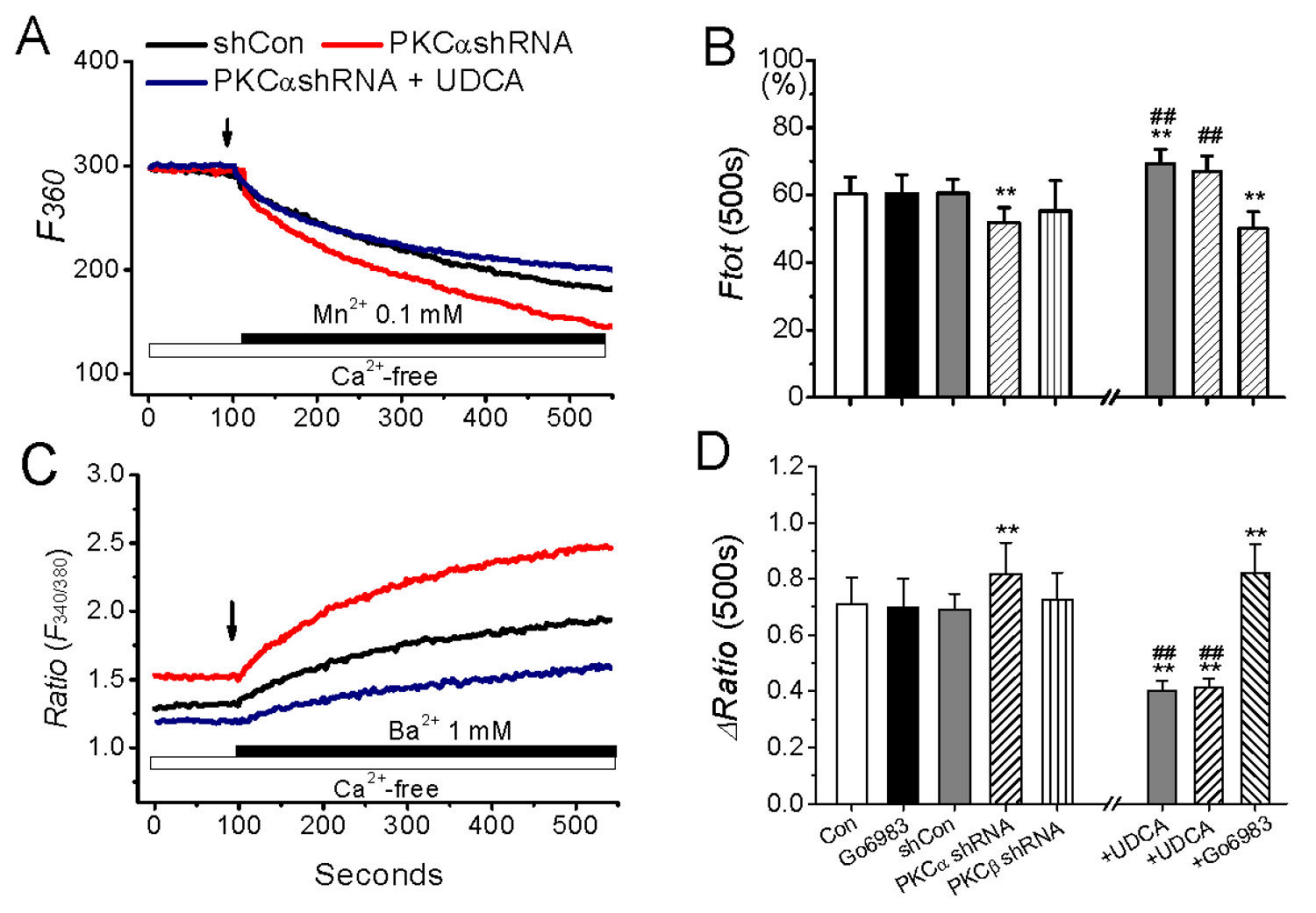
Effect of PKC interference on Mn^2+^ and Ba^2+^ influxes in resting cells. The cells transfected with PKCα and PKCβ shRNA incubated in nominally Ca^2+^-free medium or in the presence of UDCA(100 μM) for 10 min, and then were exposed to Mn^2+^ (A) or Ba^2+^ (C), respectively. The data in B and D represent the statistic results from separate groups as indicated for Mn^2+^ quench or Ba^2+^ influx protocol. **represents p<0.01 vs. the basal Mn^2+^ or Ba^2+^ influx in shCon-treated cells, and ^##^represent p<0.01 vs. those in shCon-treated or PKC shRNA-treated cells, respectively. N=8−12 independent determinations for each bar.

Here, in nominally Ca^2+^-free medium MnCl_2_ (0.1 mM) induced a resting rate of fluorescence quench, but the quench intensity was higher in PKCα-knockdown cells than those in PKCβ-knockdown and control cells (Figure 3A and B). Similarly, a basal Ba^2+^ (1 mM) influx, occurred in all cells, was also enhanced in PKCα-knockdown cells (Figure 3C and D). Blockade of PKC with Gö6983 did not affect either Mn^2+^ quench or Ba^2+^ influx, but UDCA abolished the PKC deficiency-promoted Mn^2+^ and Ba^2+^ entries (Figure 3B and D), as it did to the Ca^2+^ entry (Figure 2). 

**Figure 4:**
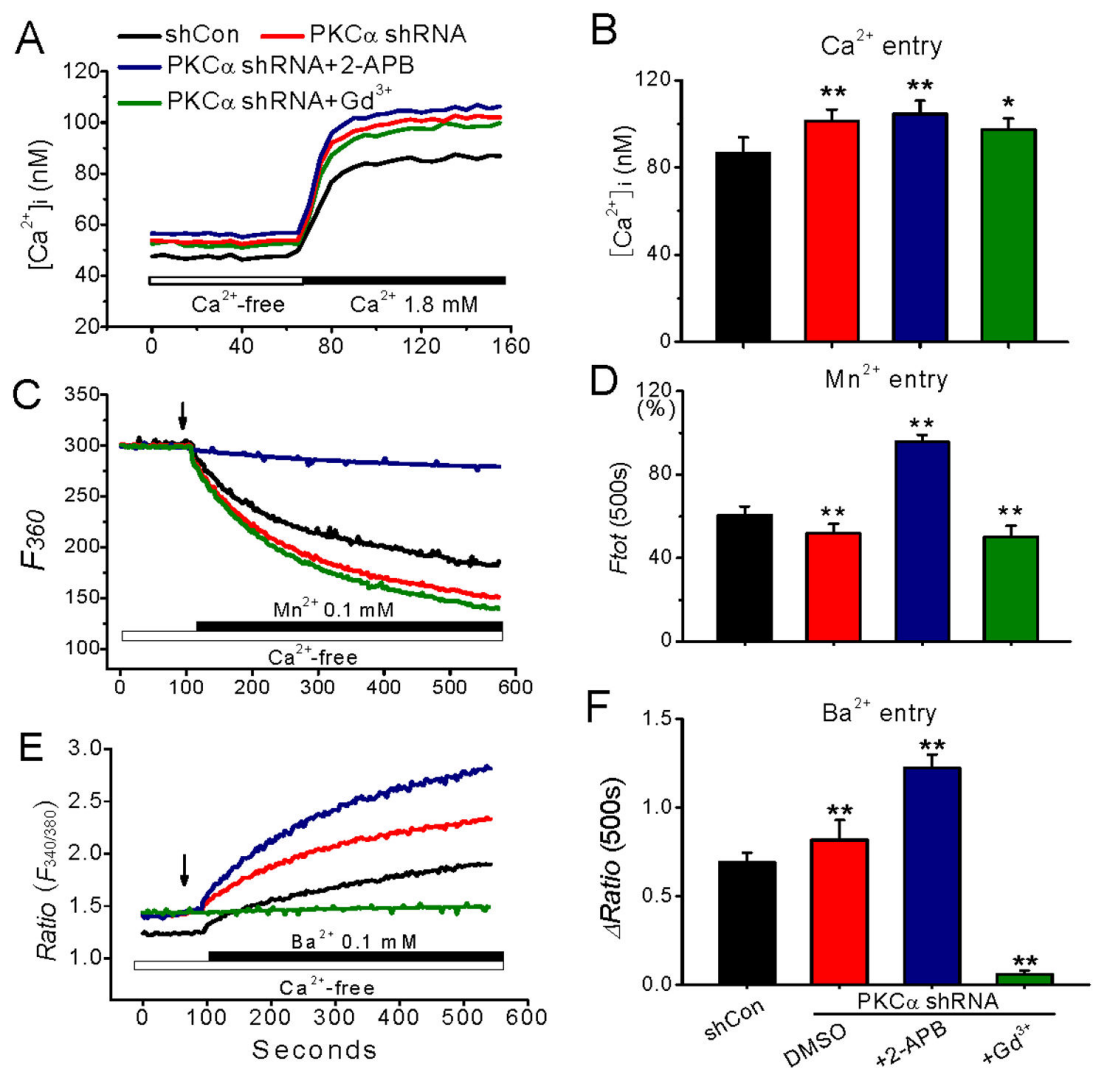
Effect of 2-APB and Gd^3+^ on divalent cations influxes in PKCα-knockdown cells. The cells transfected with PKCα shRNA incubated in nominally Ca^2+^-free medium in the presence of 2-APB (100 μM) or Gd^3+^ (10 μM) for 10 min, and then were exposed to 1.8 mM Ca^2+^ (C), 0.1 mM Mn^2+^ (D) or 1 mM Ba^2+^ (E), respectively. The data represent the statistic results from separate groups as indicated for 1.8 mM Ca^2+^, Mn^2+^ quench or Ba^2+^ influx protocol. ^*^and ^**^represents p<0.05 and p<0.01 vs. the levels detected in shCon-treated cells, respectively. N=8−12 independent determinations for each bar.

Furthermore, 2-aminoethyoxydipheyl borne (2-APB) and Gd^3+^, both are potent inhibitors of store-operated Ca^2+^ channel [20,28], were used to identify the difference of Mn^2+^ and Ba^2+^ influxes and their effects on the enhanced ion inflow in PKC-knockdown cells. As previously found [21,28], Gd^3+^ (10 μM) completely blocks the basal Ba^2+^ entry, while the basal Mn^2+^ influx is much sensitive to 100 μM 2-APB inhibition, but neither 2-APB nor Gd^3+^ could affect the PKCα deficiency-induced Ca^2+^ influx (Figure 4). These results suggest that the three divalent cation influxes that may enter cells through different channels were all augmented in PKCα-knockdown cells, and this PKC-mediated enhancement of ion inflow was not sensitive to specific antagonist but sensitive to UDCA, a membrane stabilizer. 

### 4 Effect of chronic activation of PKC on basal membrane permeability to divalent cations

**Figure 5:**
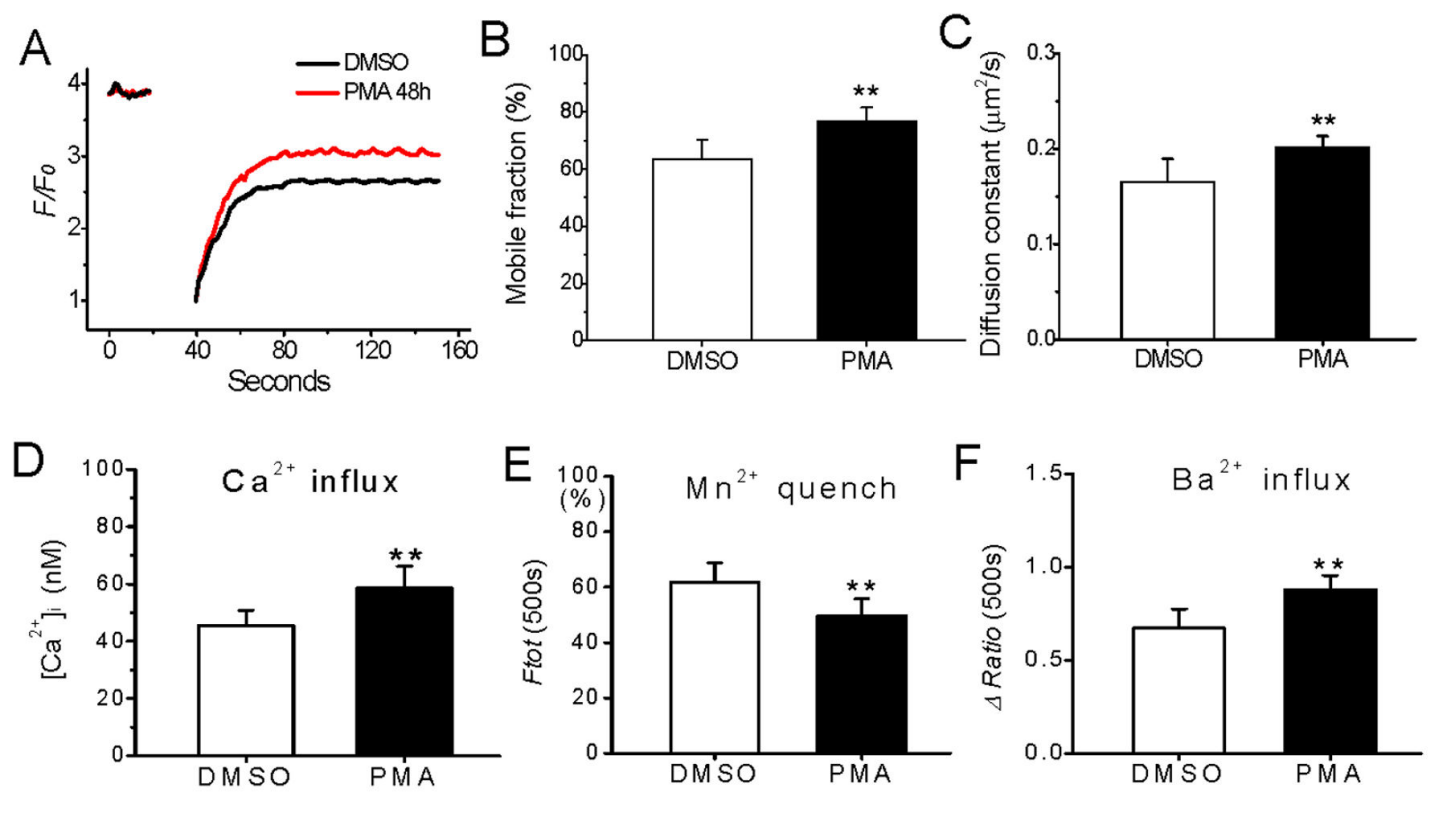
Effect of chronic activation of PKC on basal lateral membrane mobility and permeability to divalent cations. HEK293 cells were treated with 1 μM PMA for 48h, while DMSO was used as control. Then, the lateral membrane mobility was measured by FRAP as Figure 1, and basal [Ca2+]i , Mn2+ quench and Ba2+ influx was measured as the protocol in Figure 2 and Figure 4,respectively. The basal lateral membrane mobility increased (A−C) and permeable to divalent cations influxes enhanced (D−F) after long-termed activation of PKC. **represents p<0.01 vs. the levels detected in DMSO-treated cells. N=8−12 independent determinations for each bar.

It has long been described that the chronic treatment with phorbol myristate esters (PMA) induced downregulation of PMA-sensitive PKC expression in many types of cells [7,18,19]. Thus, we further investigated if membrane permeability to divalent cations is also altered because of the insufficient PKCs in long term PMA-treated cells. As found in PKCα knockdown cells, basal lateral membrane mobility, and Ca^2+^, Mn^2+^ and Ba^2+^ influxes were all increased in the cells treated with 1 μM PMA for 48 h compared with that in control cells (Figure 5). UDCA at a concentration of 100 μM also abolished the PMA-induced potentiation of divalent cation influxes (data not shown). 

### 5 Responses of PKCα distribution and expression to short and long-term PKC activation

**Figure 6:**
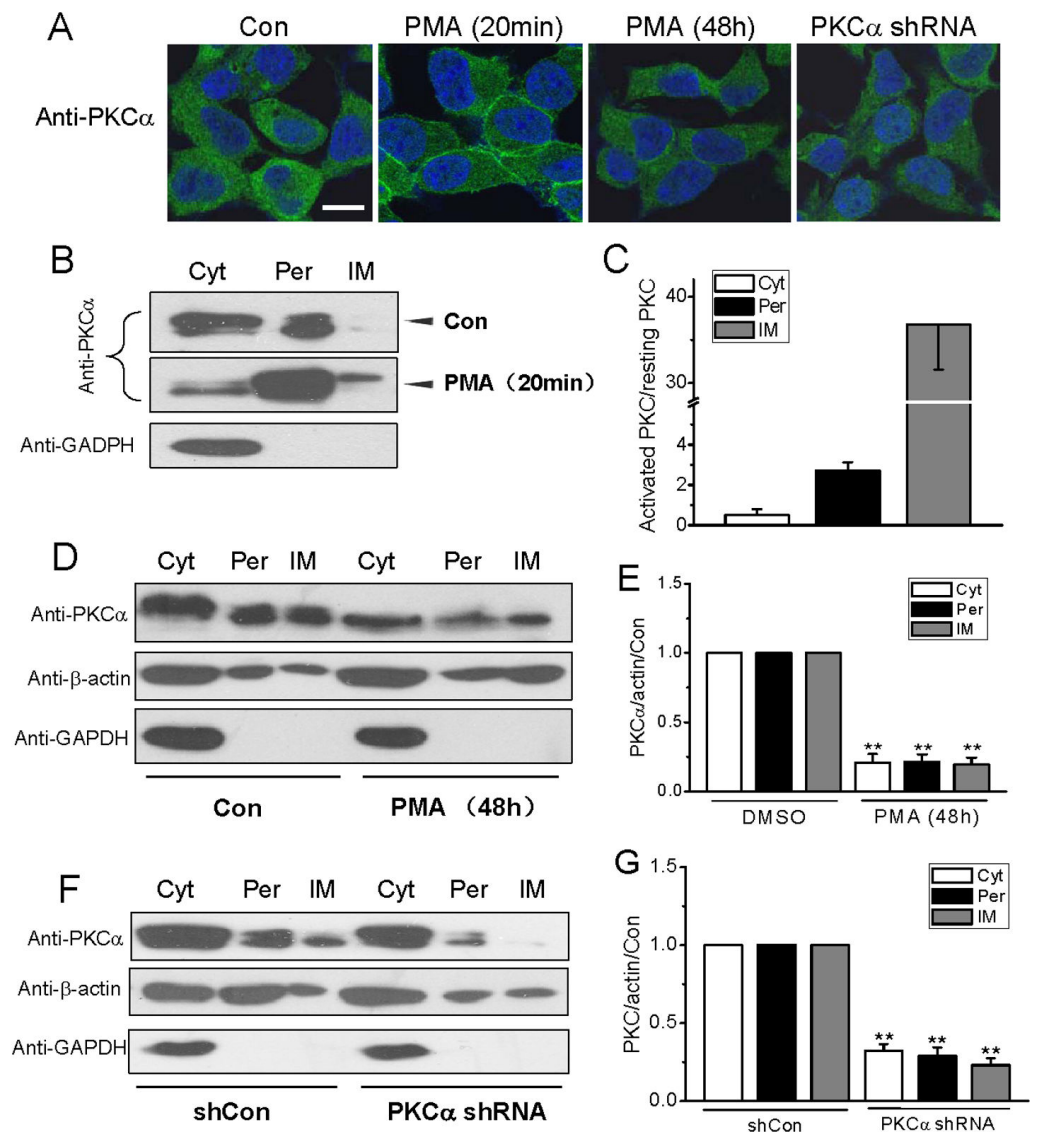
Responses of PKCα distribution and expression to short and long-term PKC activation. In (A), the subcellular distribution of PKCα was detected by immunocytochemical staining of HEK293 with antibodies specific for PKCα, and nucleus were labeled with Hoechst 33258 (1 μg/ml), scale bar: 10 μm; (B and C), cells were treated with PMA (1 μM) for 20 min, and subcellular fractionations were obtained by ultracentrifugation and Triton X-114 phase partitioning. Then ~2% of the cytosolic protein, ~2% of the peripheral membrane protein and ~5% of the integral membrane protein fractions were loaded for Western analysis respectively. Cyt, represents cytosolic proteins; Per, peripheral membrane proteins; and IM, integral membrane proteins. The data, indicating quantification of the change in mass of subcellular fractions of PKCα, are expressed as fold changes of each fraction after PMA-treatment. (D and E), the cells were treated with PMA (1 μM) for 48 h, and each of the fractions were loaded for Western analysis with 15 μg for each sample. The long-termed activation of the PKC induced a significant reduction in PKCα expression. (F and G), each of PKCα fractions was loaded with 15 μg for each sample. The expressions of PKCα in three fractions were decreased equally and the levels of reductions were similar with the whole cells lysate. Western blotting for each sample were performed in three separate experiments ^**^represent p<0.01*vs*. the PKC expression in DMSO-treated or shCon-treated cells, respectively.

Finally, we investigated the changes of PKCα distribution and expression in different fractions of cell lysates by Western blot to identify the relationship between the membrane distributed-PKCs and regulation of membrane permeability. Successful separation of membrane from cytosol was demonstrated by the fact that GAPDH, a cytosolic protein often used as a loading control for Western blotting detection, was absent from the membrane fraction, and levels of β-actin were used as normalizing controls in each fraction (Figure 6B, D and F).

**Figure 7:**
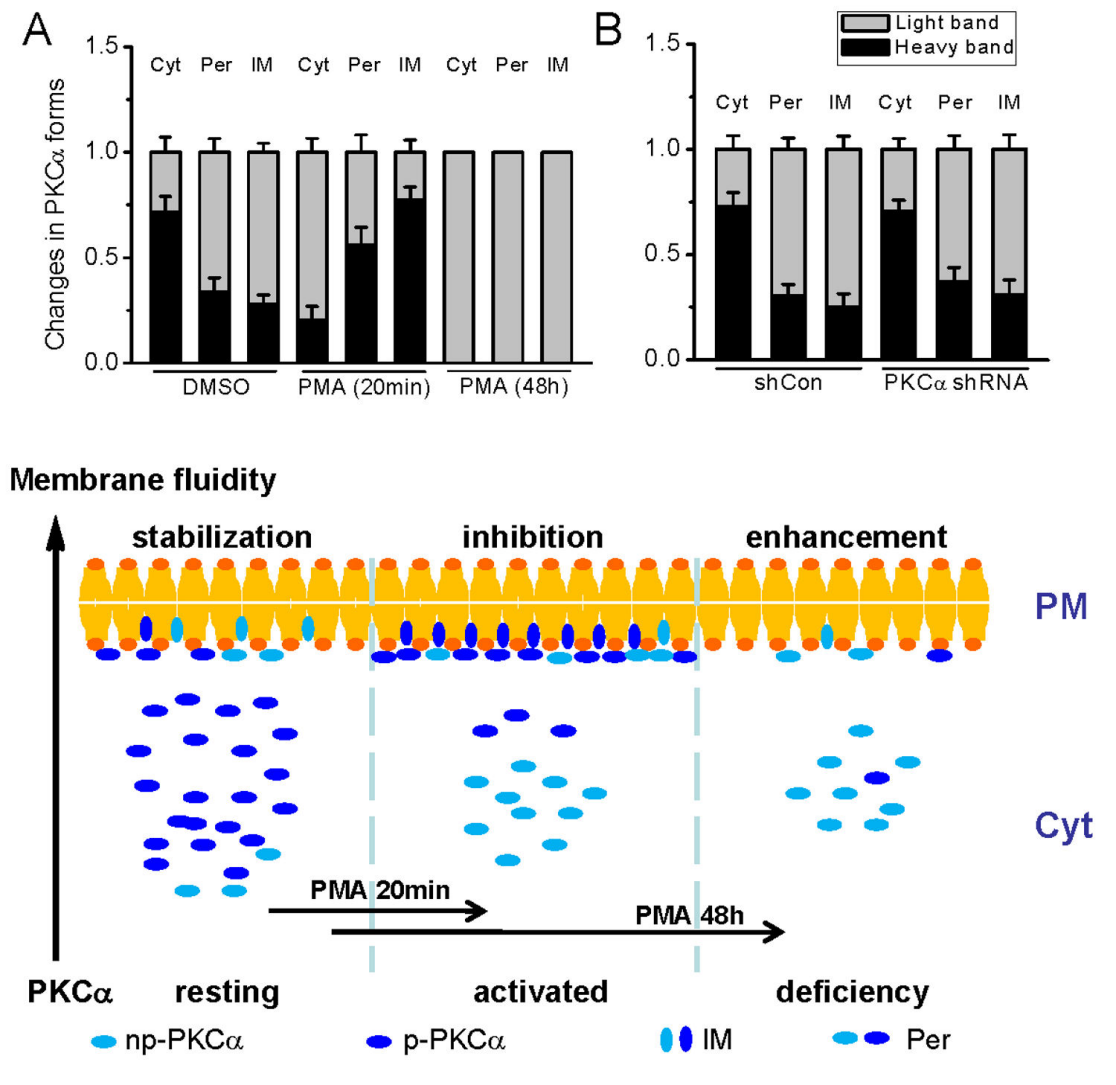
Analysis of blots and the schematic diagram of PKCα distribution and its effect on membrane. (A and B), total band intensities in three fractions were considered as 1, and the percentage of the light band (non-phosphorylated forms) and the heavy band (phosphorylated forms) were analyzed in three separate experiments. Schematic diagram, the PKCα was divided into two groups as “np-PKCα” (non-phosphorylated forms) and “p-PKCα” (phosphorylated forms). Per, represents peripheral membrane proteins; and IM, integral membrane proteins. The proportion and distribution of the two forms of PKCα were changed due to short/long term activation by PMA. The level of membrane-associated PKCα is inversely proportional to lateral membrane mobility.

In shCon RNA- and DMSO-treated cells, the respective PKCα proportions distributed in cytosolic, peripheral membrane and integral membrane pools were 80.99%, 18.78% and 0.23%, generally in agreement with the finding in NIH3T3 cells [7]. As expected, a dramatic aggregation of PKCα in plasma membrane was induced by activation of PKC with PMA for 20 min, but parallel reductions were found in all the three pools after activation with PMA for 48 h (Figure 6A–E) or PKCα knockdown with shRNA plasmid (Figure 6F and G). The integral and peripheral membrane PKCα pools increased approximately 40 folds from 0.23% to 8.56% and 2.6 folds from 18.78% to 50.88%, respectively, while the cytosolic PKCα concomitantly decreased to half from 80.99% to 40.55% upon PMA stimulation for 20 min (Figure 6C). In contrast, approximate 80% or 70% reductions in PKCα expressed in all three pools were found in cells exposure to PMA or PKCα silencing gene for 48 h (Figure 6G). Additionally, it is also notable that obvious shifts in two PKCα bands, the light band (non-phsophorylated forms) and the heavy band (phosphorylated forms) [29–31], occurred in the cells stimulated with PMA (Figures 6, and 7A and B). In cells activated with PMA for 20 min, the phsophorylated PKCα form was much more in both membrane-associated fractions but less in cytosol pool than those in control cells. Contrarily, there appeared no detectable phosphorylated form in all pools after PMA stimulation for 48 h, suggesting a strong depletion of phosphorylated PKCα by chronic activation of PKC. Since phosphorylated and non-phosphorylated PKCα forms were accordingly lowered in all the three pools of PKCα knockdown cells (Figure 7A and B), the common effect of interfering PKC by shRNA and persistent stimulation with PMA on basal membrane stabilization is likely irrespective of PKC (de)phosphorylation states.

Taken together, these data here showed that the lateral membrane mobility is intimately related with the level of membrane-associated PKCα that is determined integrally by the PKC dynamic recycling activity (Figure 7C).

## Discussion

This study demonstrates a novel effect of PKC on basal lateral membrane mobility that is associated with the maintenance of ion influx across the plasma membrane in resting cells, and PKCα is the most responsible isotype for this regulatory effect. This conclusion is based mainly on the following observations: i) the basal lateral membrane mobility in resting cells increases in response to PKCα impairment by either knocking down PKCα expression or long-term activation of PKC (Figures 1 and 5); ii) basal divalent cations influxes, which appear through different channels in plasma membrane (Figure 4), are also accordingly augmented (Figures 2, 3 and 5); iii) the membrane stabilizer UDCA abolishes both the changes of lateral membrane mobility and permeability to ions due to insufficient PKCα, whereas Gö6983 to block both PKCα and PKCβ activation does not affect the lateral membrane mobility and the altered membrane permeability induced by PKC deficiency (Figures 1−3), suggesting that interference of PKC disturbs membrane stabilization, and this perturbation in the membrane is independent of endogenous PKC activation; and iv) the significant increases in the lateral membrane mobility and divalent ion fluxes are accompanied by the downregulation of membrane PKCα expression (Figure 6), whereas short-term activation of PKC with PMA invokes large amount of PKCα aggregation in the plasma membrane (Figure 6) with both downregulated membrane fluidity and Ca^2+^ influx [13], indicating an intimate link between the level of PKCα and the regulation of membrane fluidity in plasma membrane (Figure 7). 

It is generally accepted that the membrane fluidity can affect the functions of a number of membrane-bound enzymes, ionic channels and receptors, including those present in the endoplasmic reticulum [28,32]. In the previous studies [13,14], we found that the conventional PKCs accumulate in plasma membrane and endoplasmic reticulum and, as a result, downregulate the membrane fluidities and Ca^2+^ fluxes upon cell activation. The present study further extends to that the native membrane-associated PKCα is involved in the maintenance of basal membrane stabilization/permeability in resting cells. In previous studies [13,14], inhibition of PKC activation with Gö6983 abolishes the redistribution of PKCs and their regulatory effects on the plasma membrane and endoplasma membrane fluidity and permeability to Ca^2+^ when cells are stimulated. Here, unlike that previously, found this effect of PKC in resting cells does not require the activation of the kinase because Gö6983 showed no effect on the PKC-associated membrane stabilization, whereas PKC deficiency affected such effect in resting cells. This suggests that the preexisted native PKCs in the cell membrane [7] may take their responsibility for the maintenance of basal membrane homeostasis, and the robust translocations of PKCs further enhance this effect to protect cells against exaggerated change in membrane fluidity upon activation. 

Such regulatory role of PKC in basal lateral membrane mobility is probably much important for the basic cellular functions and homeostasis because normal membrane fluidity/permeability confers cells to survival and to resist the environmental perturbations [33,34]. It has been found that the expressions of PKCα are decreased in erythrocyte in elderly hypertensives [35] and in hippocampus of aged rabbit [36], while upregulation of PKCα ameliorates age-related neuroplasticity [37], implying a linkage of PKC with the age-related disease pathogenesis. Additionally, hepatic apoptosis at early and late phases of polymicrobial sepsis and ethanol-induced hepatic oxidative stress have been found related to the decreases of PKCα expression [38] and membrane fluidity [16] in hepacytes. More consistent with the present results, PKC-depleted macrophages by chronic exposure to PMA exhibit an approximately 40% lower membrane microviscosity and more uptake of parasite than normal macrophages [39]. Therefore, all these reports indicate an important role of PKC in maintenance of basal cellular homeostasis, in particular the lateral membrane mobility in resting cells, and an elevation in membrane permeability is more or less involved in the disturbance of cellular functions and also disease development.

Biological membranes in general consist of various lipids and sterols, which amount to about 50% by mass, the other half being constituted by membrane proteins. Large amount of membrane proteins are generally permanently located in the membrane, forming channels and transporters for molecules’ exchanges between the interior and exterior of the cell, while small amount of membrane proteins including the conventional PKCs are trafficking between the membrane and cytoplasm for signal transduction upon cell activation. It is well recognized that PKCα is synthesized as a soluble unphosphorylated protein initially, and accumulates phosphates at three priming sites: Thr^497^, Thr^638^ and Ser^657^ during conformation processing. The fully phosphorylated PKCα localizes to cytosol, and is recruited to membranes and activated by binding with Ca^2+^ and diacylglycerol to ignite the downstream protein activations upon cell stimulation. The inactive PKCα form is relatively resistant to dephosphorylation and degradation, but the membrane-bound conformations are more easily to be dephosphorylated, an unstable form shunted to degradation [29,40]. Thus, long-term activation of PKC with PMA results in more degradation of phosphorylated form and ultimately depletion of total PKCs (Figure 6D). In the present study, the non-phosphorylated species (light band), including dephosphorylated and newly synthesized unphosphorylated PKCα, and the phosphorylated forms (heavy band), including active and inactive PKCα, are detectable in all the three fractions of cell lysates (Figure 6). However, PKCα-associated regulation of membrane permeability is probably independent of either form of PKCs (Figure 7A and B), instead, the level of PKCα in the plasma membrane is a crucial factor, likely resembling the membrane stabilization effect of bovine serum albumin [41,42]. Actually, several studies have revealed that the membrane proteins, especially the integral proteins, are able to influence the cell membrane permeability [43,44]. 

In summary, the endogenous PKCs sited in the plasma membrane in resting cells are involved in the maintenance of membrane characteristics, in particular, the basal lateral membrane mobility and permeability. Physiological or pathological downregulation of PKC expression is inclined to increase membrane permeability to ions, signal molecules or even harmful stresses, causing perturbations in cellular homeostasis and functionality that may potentially contribute to some chronic disease incidence and development [35,36,38]. 
